# A Facile machine learning multi-classification model for *Streptococcus agalactiae* clonal complexes

**DOI:** 10.1186/s12941-022-00541-3

**Published:** 2022-11-18

**Authors:** Jingxian Liu, Jing Zhao, Chencui Huang, Jingxu Xu, Wei Liu, Jiajia Yu, Hongyan Guan, Ying Liu, Lisong Shen

**Affiliations:** 1 Department of Clinical Laboratory, Xin Hua Hospital, Shanghai Jiao Tong University School of Medicine, 1665 Kong Jiang Road, Shanghai, 200092 China; 2 Department of Research Collaboration, R&D Center, Beijing Deepwise & League of PHD Technology Co., Ltd, Beijing, 100080 China; 3 Faculty of Medical Laboratory Sciences, Shanghai Jiao Tong University School of Medicine, Shanghai, 200001 China

**Keywords:** *Streptococcus agalactiae*, Multilocus sequence typing, Clonal complex, Machine learning

## Abstract

**Background:**

The clinical significance of group B streptococcus (GBS) was different among different clonal complexes (CCs), accurate strain typing of GBS would facilitate clinical prognostic evaluation, epidemiological investigation and infection control. The aim of this study was to construct a practical and facile CCs prediction model for *S. agalactiae*.

**Methods:**

A total of 325 non-duplicated GBS strains were collected from clinical samples in Xinhua Hospital, Shanghai, China. Multilocus sequence typing (MLST) method was used for molecular classification, the results were analyzed to derive CCs by Bionumeric 8.0 software. Antibiotic susceptibility test was performed using Vitek-2 Compact system combined with K-B method. Multiplex PCR method was used for serotype identification. A total of 45 virulence genes associated with adhesion, invasion, immune evasion were detected by PCR method and electrophoresis. Three types of features, including antibiotic susceptibility (A), serotypes (S) and virulence genes (V) tests, and XGBoost algorithm was established to develop multi-class CCs identification models. The performance of proposed models was evaluated by the receiver operating characteristic curve (ROC).

**Results:**

The 325 GBS were divided into 47 STs, and then calculated into 7 major CCs, including CC1, CC10, CC12, CC17, CC19, CC23, CC24. A total of 18 features in three kinds of tests (A, S, V) were significantly different from each CC. The model based on all the features (S&A&V) performed best with AUC 0.9536. The model based on serotype and antibiotic resistance (S&A) only enrolled 5 weighed features, performed well in predicting CCs with mean AUC 0.9212, and had no statistical difference in predicting CC10, CC12, CC17, CC19, CC23 and CC24 when compared with S&A&V model (all *p* > *0.05*).

**Conclusions:**

The S&A model requires least parameters while maintaining a high accuracy and predictive power of CCs prediction. The established model could be used as a promising tool to classify the GBS molecular types, and suggests a substantive improvement in clinical application and epidemiology surveillance in GBS phenotyping.

**Supplementary Information:**

The online version contains supplementary material available at 10.1186/s12941-022-00541-3.

## Background

*Streptococcus agalactiae,* also named group B streptococcus (GBS) is a dynamic colonizer of the gastrointestinal and genitourinary tracts. However, it is a leading cause of neonatal and maternal invasive diseases. And recently, the GBS infection rate in non-pregnant adults was also reported increasing rapidly [1]. It is known that different GBS clones present different features on pathogenicity. CC17 is considered as a hypervirulent clone, which could easily transfer to newborns through maternal–fetal transmission or other ways, and cause severe infections such as sepsis and meningitis [2, 3]. Other clones such as CC19 have been reported mostly associated with carriage [4]. An accurate prediction for molecular epidemiology information of the clinical isolated GBS is therefore needed.

Previously, the multilocus sequence typing method (MLST) result was proved associated with serotyping, and some reports showed that the antibiotic resistance profile was also related to sequence type (ST)s [5, 6]. The main strategy to learn the molecular epidemiology of GBS is MLST. It needs to amplify, sequence and blast 7 house-keeping genes [7], which required highly-trained personnel and expensive equipment, and is time-consuming. Moreover, it's mostly used as a retrospective epidemiological investigation tool but not implemented into clinical application. A more facile, economical, clinically available method for GBS classification is required.

Currently, in the field of medical laboratory, machine learning is a viable, powerful tool to support clinical decision making and microorganism classifying. Wang et al. [8] construct a prediction model for five different serotypes (Ia, Ib, III, V, VI) of GBS based on Matrix-assisted laser desorption ionization-time of flight mass spectrum (MALDI TOF MS) and machine learning. A previous study also generated a predictive model for ST5, ST59, ST239 and ST45 of methicillin-resistant *Staphylococcus aureus* (MRSA) strain through machine learning methods [9]. However, no available model for GBS STs or CCs prediction had been constructed yet.

Therefore, we aim to develop a machine learning-based multi-class classification model to assist for classifying different CCs of GBS, using three kinds of laboratory test features, including antibiotic susceptibility test (A), serotypes test (S) and virulence genes(V) test. To achieve this, we adopted machine learning models that could be used to differentiate the molecular types of GBS and easily used in clinical strategy implement and epidemiology surveillance.

## Methods

### Isolate collection

A total of 325 GBS strains isolated from clinical samples in Xinhua Hospital, Shanghai Jiao Tong University were enrolled in this study. The isolates were stored at -80℃ in glycerin broth, then recovered and cultured onto 5% sheep blood plate for 24 h at 37 ℃ in 5% CO_2_ atmosphere, and re-identified by MALDI-TOF MS (Microflex™ LT, Bruker Daltonik, Germany).

### MLST

MLST was conducted by sequencing seven housekeeping genes, *adhP*, *pheS*, *atr*, *glnA*, *sdhA*, *glcK* and *tkt* as previously described [7]. The sequence type was determined via *S. agalactiae* MLST database (https://pubmlst.org/sagalactiae/). New alleles or ST profiles were submitted and assigned at the *S. agalactiae* MLST database. Bionumeric 8.0 software was used for homology analysis, a founder ST and its single locus variates (SLVs) were defined as a clonal complex (CC). The CCs were named after the founder STs.

### Antibiotic susceptibility test

Susceptibility to penicillin G, ampicillin, vancomycin, erythromycin, clindamycin, levofloxacin, ceftriaxone, tetracycline and linezolid was measured by Vitek-2 Compact system combined with Kirby–Bauer’s disk diffusion (KB) method according to the Clinical and Laboratory Standard Institute standards (CLSI, 2020). *S. pneumoniae* ATCC49619 was used as a control strain.

### Serotyping

The nine GBS serotypes (Ia, Ib, II–VIII) based on capsular polysaccharide (CPS) were distinguished using multiplex PCR method developed previously [10]. Strains not belong to any of the above nine serotypes were submitted to a serotype IX-specific PCR as described by Kong F et al. [11]. Non-typeable isolates were designated as NT.

### Virulence genes

Forty-five virulence genes associated with adhesion, invasion and immune evasion were detected by PCR method. Primers and amplification condition were previously described [12]. The PCR products were visualized by agarose gel electrophoresis with SYBR safe gel stain.

### Machine learning

Deepwise & Beckman Coulter DxAI platform (https://dxonline.deepwise.com/) was used to construct CC prediction models. CCs were categorized into 7 major categories (CC1, CC10, CC12, CC17, CC19, CC23 and CC24) and XGBoost algorithms was selected to construct predictive models. XGBoost is an ensemble method with which models are built sequentially to minimize the errors and maximize the influence of the best models. The results of antibiotic resistance, serotypes, and the virulence genes were defined as independent variables. Features had statistical difference among different CCs were selected using Chi-square test, and were classified into three major categories, antibiotic resistance (A), serotype (S), and carriage of virulence genes (V). Machine learning models were constructed by any combinations of the above categories. The dataset of GBS strains was randomly separated into a training set (70%) and testing set (30%). Statistical test of linear models penalized with the L1 norm was used for feature selection. The area under the ROC curve (AUC) was used to determine the model’s performances, and DeLong test was used to compare the AUC values between different models. For all tests, *p* value < 0.05 was considered to be statistically significant.

## Results

### The molecular epidemiology characteristic of GBS isolates

**Fig. 1:**
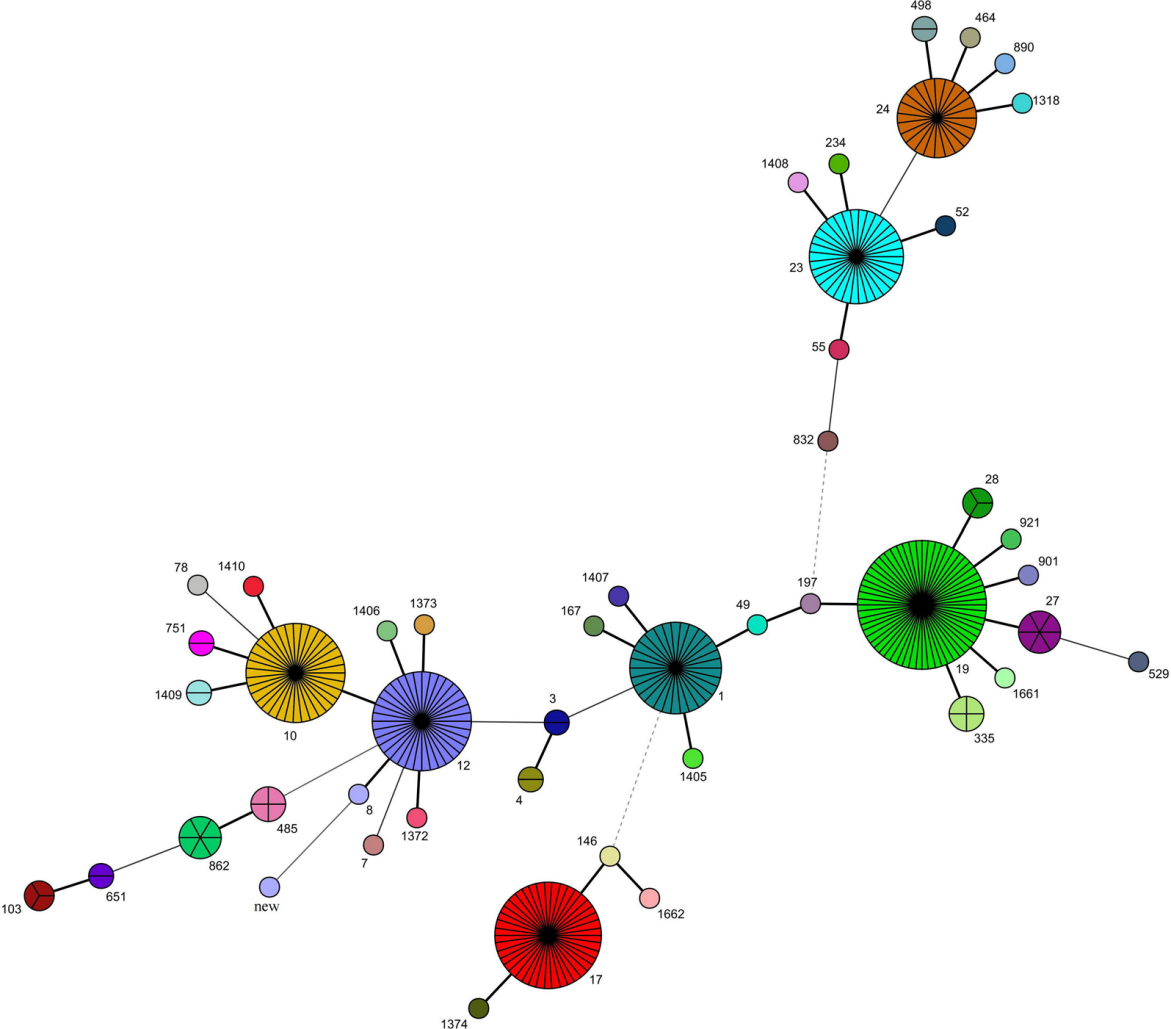
Minimum spanning tree of STs of 325 *S. agalactiae* isolates. Each node represents a single sequence type (ST), the numbers present beside the nodes are related STs. The node size is proportional to the number of isolates within the represent ST. The distance of the nodes represent the genetic relationship between STs. The single locus variates (SLVs) are connected using bold black lines

The 325 strains could be divided into 47 STs, the most common ones were ST19 (18.5%), ST17 (12.9%), ST12 (11.1%), ST10 (10.8%), ST23 (10.2%), ST1 (9.2%) and ST24 (7.1%). The MLST results were then hierarchically clustered by minimum spanning trees method, 7 major clonal complexes (CCs) were derived, including CC19 (containing ST19, ST27, ST28, ST336, ST901, ST921 and ST1661), CC17 (ST17, ST146, ST1374), CC12 (ST12, ST8, ST1372, ST1373, ST1406), CC10 (ST10, ST751, ST1409, ST1410), CC23 (ST23, ST52, ST55, ST234, ST1408), CC1 (ST1, ST167, ST1405, ST1407) and CC24 (ST24, ST454, ST498, ST890, ST1318) (Fig. 1).

### The characteristics of GBS in different CCs

All strains were susceptible to penicillin, ceftriaxone, vancomycin and tigecycline, the resistance rates of erythromycin, clindamycin, levofloxacin, and tetracycline were 79.1%, 64.0%, 34.2% and 83.1% respectively. Each CC had specific antibiotic resistance profile such as 100% CC10 strains were susceptible to tetracycline, while 95% were resistant to levofloxacin. The resistance of erythromycin, clindamycin, levofloxacin, and tetracycline were significantly different from CCs (Table 1).

**Table 1:** Characteristics of *S. agalactiae* in different CCs

			CC									*X*^*2*^	*p*
			1 (n = 33)	10 (n = 40)	12 (n = 41)	17 (n = 44)	19 (n = 76)	23 (n = 36)	24 (n = 28)	O (n = 27)	Total (n = 325)		
Antitibiotic resistance	ERY	I	0	0	6	5	16	0	1	0	28	102.620	0.000 *
		R	21	36	33	34	41	12	7	17	201		
		S	12	4	2	5	19	24	20	10	96		
	CLI	I	0	0	0	1	0	0	0	0	1	104.514	0.000 *
		R	22	35	40	33	48	7	3	18	206		
		S	11	5	1	10	28	29	25	9	118		
	LVX	I	1	0	0	0	1	2	0	0	4	226.202	0.000 *
		R	2	38	0	0	57	2	0	4	103		
		S	30	2	41	44	18	32	28	23	218		
	TCY	I	0	0	0	0	2	1	0	0	3	192.377	0.000 *
		R	25	3	41	44	69	34	26	21	263		
		S	8	37	0	0	5	1	1	6	58		
Virulence genes	*fbsA*		13	34	39	1	7	33	25	9	161	190.419	*0.000*
	*scpB*		32	40	41	44	75	36	28	11	307	163.006	*0.000*
	*lmb*		32	39	41	43	75	35	28	12	305	125.045	*0.000*
	*Gpc1*		25	1	34	19	74	0	0	6	159	203.944	*0.000*
	*Gpc2*		33	38	34	19	74	0	0	10	208	208.924	*0.000*
	*Gpc3*		33	38	34	19	74	0	0	10	208	208.924	*0.000*
	*Gpc4*		32	38	34	19	74	0	2	10	209	196.167	*0.000*
	*Gpc5*		33	38	34	19	74	0	0	10	208	208.924	*0.000*
	*cylK*		0	1	1	0	1	35	27	2	67	283.902	*0.000*
	*cfb*		33	40	41	44	76	36	28	24	322	33.420	*0.000*
	*spb1*		23	38	41	0	3	1	0	3	109	255.955	*0.000*
	*bac*		0	30	11	1	0	1	1	2	46	157.811	*0.000*
	*cpslaJ*		0	1	0	44	64	31	0	21	161	244.783	*0.000*
	*cpsG*		7	1	0	44	64	0	1	12	129	223.793	*0.000*
	*cpsI*		0	0	0	44	64	0	0	10	118	254.075	*0.000*
	*cpsJ*		0	0	0	43	62	0	0	10	115	243.235	*0.000*
Serotypes	Ia		0	0	0	0	0	30	0	11	41	838.675	*0.000*
	Ib		0	38	41	0	0	0	0	1	80		
	II		0	2	0	0	3	0	0	1	6		
	III		1	0	0	44	62	0	0	10	117		
	IV		0	0	0	0	0	1	0	0	1		
	V		19	0	0	0	9	4	28	2	62		
	VI		9	0	0	0	0	0	0	2	11		
	VII		3	0	0	0	1	0	0	0	4		
	ND		1	0	0	0	1	1	0	0	3		

*CCs* clonal complexes, *ERY* erythromycin, *CLI* clindamycin, *LVX* levofloxacin, *TCY* tetracycline

^*^P < *0.05*, the difference is significant

A total of 8 serotypes were detected, including Ia, Ib, II, III, IV, V, VI, VII, with III (36.0%), Ib (24.6%), V (19.1%), Ia (12.6%) the most frequent. The serotypes also had a correlation with CCs. All the CC12, CC17, and CC24 strains belong to serotype Ib, III and V, respectively. 95% of CC10 strains belong to serotype Ib, 81.6% of CC19 strains belong to III, 83.3% of CC23 strains belong to Ia, and 57.6% of CC1 strains belong to V. The serotype distribution among each CCs were significantly different (Table 1).

All the GBS strains were carrying virulence gene *cfb* and *pbp1A*. The positive rates of *fbsB, pavA, scpB, lmb, cylD, cylG, clyZ, cylA, cylB, cylE, cylF, cylI, cylJ, hylB, bca, neuA, neuD, neuB, cspA, cpsA, cpsB, cpsC, cpsD, cpsE, cpsF* and *cpsM* were all higher than 90%, and had no significant difference between each CCs (p > 0.05). For the rest genes, about 82.5 ~ 95.1% isolates belong to CC10, CC12, CC23 and CC24 were positive for *fbsA* gene, while only 39.4%, 9.2% and 2.3% in CC1, CC19 and CC17 GBS respectively. Almost all the CC23 and CC24 were negative for pilus cluster coding genes (*Gpc1 ~ 5*), while for CC1, CC12 and CC19 strains were with high positive rate, most of the CC10 strains were positive for *Gpc2 ~ 5*, but lack of *Gpc1* gene. More than 96% CC23 and CC24 strains carrying *cylK* gene, while rare in other CCs. The *spb1* gene was specific in CC10 and CC12 strains, *cpsIaJ* was specific in CC17, CC19, CC23, *cpsG, cpsI* and *cpsJ* were specific in CC17 and CC19. The positive rate of following genes: *fbsA, scpB, lmb, gpc1 ~ 5, cylK, cfb, spb1, bac, cpsIaJ, cpsG, cpsI, cpsJ* was distinguishable from each CCs (Table 1).

### CCs prediction model with machine learning

**Fig. 2:**
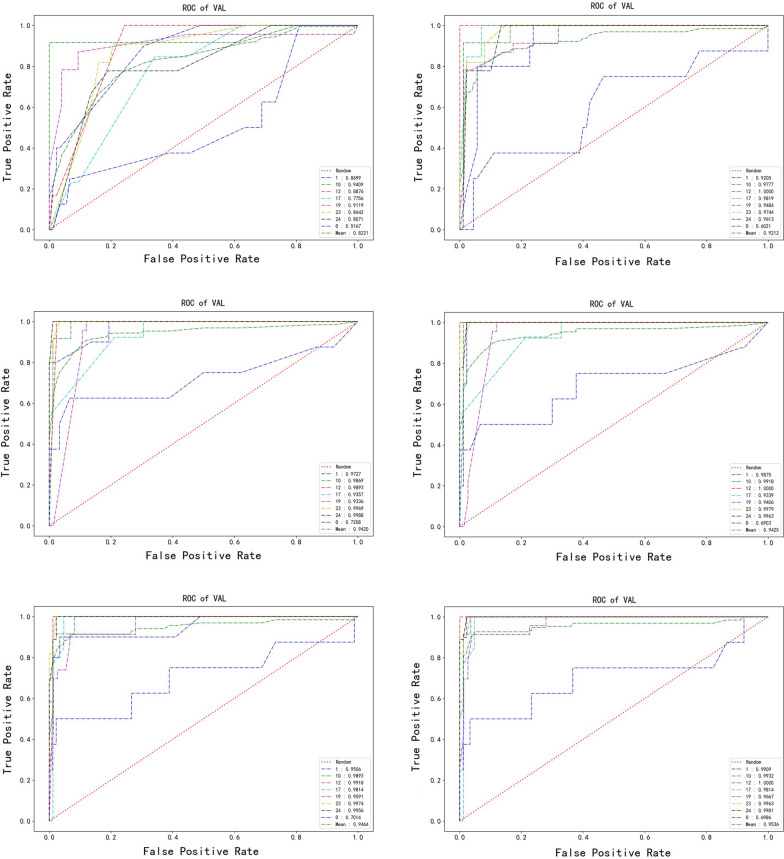
The area under the curve (AUC) of models based on different combination of features in prediction clonal complexes (CCs) of *S. agalactiae* using XGBoost algorithm. From top left to bottom right: A (the parameters include antibiotic resistance only), S&A (serotypes and antibiotic resistance), V (virulence genes detection results only), S&V (serotypes and virulence genes), A&V (antibiotic resistance and virulence genes) and S&A&V (serotypes and antibiotic resistance and virulence genes) model

A total of six CCs prediction models [antibiotic resistance only (A), virulence genes only (V), serotypes plus antibiotic resistance (S&A), serotypes plus virulence genes (S&V), antibiotic resistance plus virulence genes (A&V), serotypes plus antibiotic resistance plus virulence genes (S&A&V)] were constructed. The mean AUC of six models in a descending order is: S&A&V (0.9536) > A&V (0.9464) > S&V (0.9425) > V (0.9420) > S&A (0.9212) > A (0.8221) (Fig. 2). The A model performed worst, and could poorly predict CC17 (AUC = 0.7756). The S&A&V, A&V, S&V and V models performed well, but enrolled 16 (serotypes, resistance of four antibiotics and PCR result of *lmb*, *Gpc1*, *cfb*, *cpsIaJ*, *bac*, *cylK*, *cpsI*, *Gpc2*, *fbsA*, *scpB*, *spb1*genes), 15 (resistance of four antibiotics, PCR result of *lmb*, *Gpc1*, *cfb*, *cpsIaJ*, *bac*, *cylK*, *cpsI*, *Gpc2*, *fbsA*, *scpB* and *spb1*genes), 12 (serotypes and PCR result of *lmb*, *Gpc1*, *cfb*, *cpsIaJ*, *bac*, *cylK*, *cpsI*, *Gpc2*, *fbsA*, *scpB*, *spb1*genes) and 11 (PCR result of *lmb*, *Gpc1*, *cfb*, *cpsIaJ*, *bac*, *cylK*, *cpsI*, *Gpc2*, *fbsA*, *scpB*, *spb1*genes) parameters respectively.

The S&A model only enrolled 5 independent variables including serotypes and resistance of ERY, CLI, LVX and TCY. Feature weights of the XGBoost model were provided in Additional file 1: Figure S1. The S&A model had no statistical difference in predicting CC10, CC12, CC17, CC19, CC23 and CC24 when compared with S&A&V, A&V, S&V or V model (*p* < *0.05*) (Additional file 1: Table S1). The AUC of S&A model in predicting CC1, CC10, CC12, CC17, CC19, CC23 and CC24 was 0.9205, 0.9777, 1, 0.9819, 0.9484, 0.9744 and 0.9613. The model could distinguish the hypervirulence clone CC17 effectively, with sensitivity and specificity 0.8462 and 0.9647 respectively (Table2).The S&A prediction model based on XGBoost algorithm was available at the following website: https://dxonline.deepwise.com/prediction/index.html?baseUrl=%2Fapi%2F&id=5027&topicName=undefined&from=share.

**Table 2:** Effectiveness evaluation results of S&A CCs prediction model

Index	AUC	Accuracy	precision	sensitivity	specificity	PPV	NPV
CC1	0.9205	0.9184	0.5833	0.7	0.9432	0.5833	0.9651
CC10	0.9777	0.9694	0.8462	0.9167	0.9767	0.8462	0.9882
CC12	1	1	1	1	1	1	1
CC17	0.9819	0.949	0.7857	0.8462	0.9647	0.7857	0.9762
CC19	0.9484	0.9388	0.9474	0.7826	0.9867	0.9474	0.9367
CC23	0.9744	0.9082	0.5625	0.8182	0.9195	0.5625	0.9756
CC24	0.9613	0.9592	0.7778	0.7778	0.9775	0.7778	0.9775
Others	0.6021	0.8878	0	0	0.9667	0	0.9158
Mean	0.9212	0.7653	0.6879	0.7302	0.9669	0.6879	0.9669

*CCs* clonal complexes, *AUC* area under the curve, *PPV* positive predictive value, *NPV* negative predictive value

^*^S&A model, the CCs prediction model based on serotype and antibiotic resistance of *S. agalactiae*

## Discussion

*S. agalactiae* is a gram positive, β-hemolytic streptococcus that colonizes the urogenital and gastrointestinal tract of healthy individuals, the colonization rate is about 15%-24% in rectum,12%-17% in vagina and 5%-18% urethra, respectively [13]. However, *S. agalactiae* could cause severe invasive infection in neonatal patient through mother-to-fetus transmission, and in recent years, GBS caused adult invasive infection was reported increasing due to bacterial translocation [14, 15]. Molecular epidemiology surveillance revealed that the pathogenicity of GBS is significantly different between each clonal complex (CC) [16–19], and it is critical to distinguish different CC types of GBS strains isolated from clinical samples. By now, the GBS strains isolated from GBS screening of pregnant women, or other clinical samples, were not reported molecular typing results, since the major molecular typing method MLST was very tedious [7]. The basic procedures of MLST method include PCR, electrophoresis, purification, sequence and blast. For primary medical or scientific research institution with no sequenator, it was impossible to do this work. A facile method that could accurately classify CC types of GBS strains is necessary for efficient medical decision.

Associations between CCs and serotypes have been reported in the literature, with some suggesting a strong correlation, such as most of CC17 strains belong to serotype III, while most of CC23 strains belong to serotype Ia [5]. In this study, we found similar relationship between CCs and serotypes. In recent years, some researches revealed that the characteristic of antibiotic resistance was also associated with molecular types. Zhang etc [20] discovered that Ib/ST23 GBS strains had higher levofloxacin resistance rate than other GBS strains. Some studies reported that the ST17 and ST19 had higher tetracycline resistant rate [21]. Our findings were consistent with previous studies. We discovered that all the CC12 and CC17 strains were susceptible to levofloxacin, while most of CC10 and CC19 were resistant, almost all the CC10 strains were susceptible to tetracycline, while most of other strains were resistant. Some researches had reported the associations between serotype and virulence genes such as *cylK*, *bac*, *cylB*, *rib* and *lmb* etc [6, 12]. Few previous studies have discussed the association of MLST and virulence gene, but our research revealed that the virulence profile was quite different from each CCs. Based on the result of this study and the existing literature, we established that a CC prediction model enrolled the weighted features including antibiotic resistance profiles, serotypes, virulence gene profiles may be functional. The result of antibiotic resistance could be obtained from clinical laboratory routine process, while serotype and virulence genes could be detected by simple PCR method. The obtained results in this study using machine learning models based on above features would significantly reduce the workload and cost in molecular typing while achieved a comparable accuracy comparing to MLST method.

Artificial intelligence is prominent in the field of medical diagnosis with extensive application nowadays [22]. Recently, artificial intelligence is being increasingly applied into molecular typing of clinical isolates. Wang etc [9] had constructed a model for ST prediction of MRSA based on matrix-assisted laser desorption ionization time-of-flight mass spectrometry (MALDI TOF MS) by using machine learning approach. It is rapid but the sensitivity and specificity of classification results are relatively low. Besides, not all the institution could afford an MALDI TOF MS. In this study, we constructed a CC prediction model based on the factors which related to GBS CCs and applied machine learning technique. The model we developed extended previous study results. Our established model included only 5 covariates (i.e., the susceptibility results of four antibiotics and serotype) could accurately identify the seven common CCs. In another word, we can use the antibiotic susceptible test results achieved from routine clinical process and an additional multiple PCR amplification and electrophoresis tests to classify the isolate into CCs correctly. For some basic medical institute without experimental equipment such as PCR amplifier and electrophoresis apparatus, latex agglutination assay with a Group B streptococci typing antisera kit could be substituted [12]. In this study, we developed a simple S&A model using machine learning algorithm that does not require costly equipment and could be extensively carried out in primary medical institutions. The high AUC value of the S&A model suggested that we could employ the model to accurately categorize common CCs in laboratory settings.

Machine learning methods provide the possibility of discovering relationships that are not hypothesis driven and without prior assumptions, and indicates an innovative approach in constructing molecular typing methods. This may provide further explorations on identifying relevant biomarkers to predict CCs.

There were several limitations with our current study. First, although the study obtained a high predictive power, it is a monocentric study. Results of serotypes, antibiotic resistance and virulence gene may be biased and the classification accuracy for GBS CC types might differ if applied in other regions or countries. Further research should enroll multiple medical centers and obtain more GBS isolates to improve the generalizability of the model. Second, it would be worthwhile to finely tune the parameters and test more algorithms to allow for a better predictive system. In future work, the proposed model would collect multicenter data and include more specific features and algorithms to verify the extrapolation of the prediction models.

## Conclusion

In conclusion, we developed a machine learning-based multi-class classification model which was facile and applicable in classifying different CCs of GBS, including only 5 covariates which are results of susceptibility of four antibiotics and serotype. The XGBoost model could be used as a promising tool to accurately classify the GBS molecular types and be widely applied as an alternative method for epidemiology surveillance of GBS in regions with limited medical and research resources.

## Supplementary Information

**Additional file 1: Figure S1.** The distribution of feature weights in S&A model. The S&A model was constructed based on antibiotic resistance and serotypes of *S. agalactiae* for clonal complexes prediction*.* Characteristics with higher weight having a greater effect on the model. **Table S1.** Comparison of models based on different combination of features in three categories in predicting each CCs of *S. agalactiae.*

## Data Availability

The data and materials that support the findings of this study are available from the corresponding author [Lisong Shen] or the first author [Jingxian Liu], upon reasonable request.

## Abbreviations

AUC: Area under the curve
CC: Clonal complex
CLI: Clindamycin
ERY: Erythromycin
GBS: Group B *Streptococcus*
LVX: Levofloxacin
MALDI TOF MS: Matrix-assisted laser desorption ionization time-of-flight mass spectrometry
MLST: Multilocus sequence typing
PCR: Polymerase chain reaction
ROC: Receiver operating characteristic
ST: Sequence type
TCY: Tetracycline
XG Boost: Extreme gradient boosting
