# Supplementary material for: A Facile machine learning multi-classification model for Streptococcus agalactiae clonal complexes
Source: Ann Clin Microbiol Antimicrob. 2022 Nov 18;21:50. doi: 10.1186/s12941-022-00541-3 (PMC9675200; doi:10.1186/s12941-022-00541-3)
Supplement: Supplementary file 1 — Additional file 1: Figure S1. The distribution of feature weights in S&A model. The S&A model was constructed based on antibiotic resistance and serotypes of S. agalactiae for clonal complexes prediction. Characteristics with higher weight having a greater effect on the model. Table S1. Comparison of models based on different combination of features in three categories in predicting each CCs of S. agalactiae. [file 12941_2022_541_MOESM1_ESM.docx]

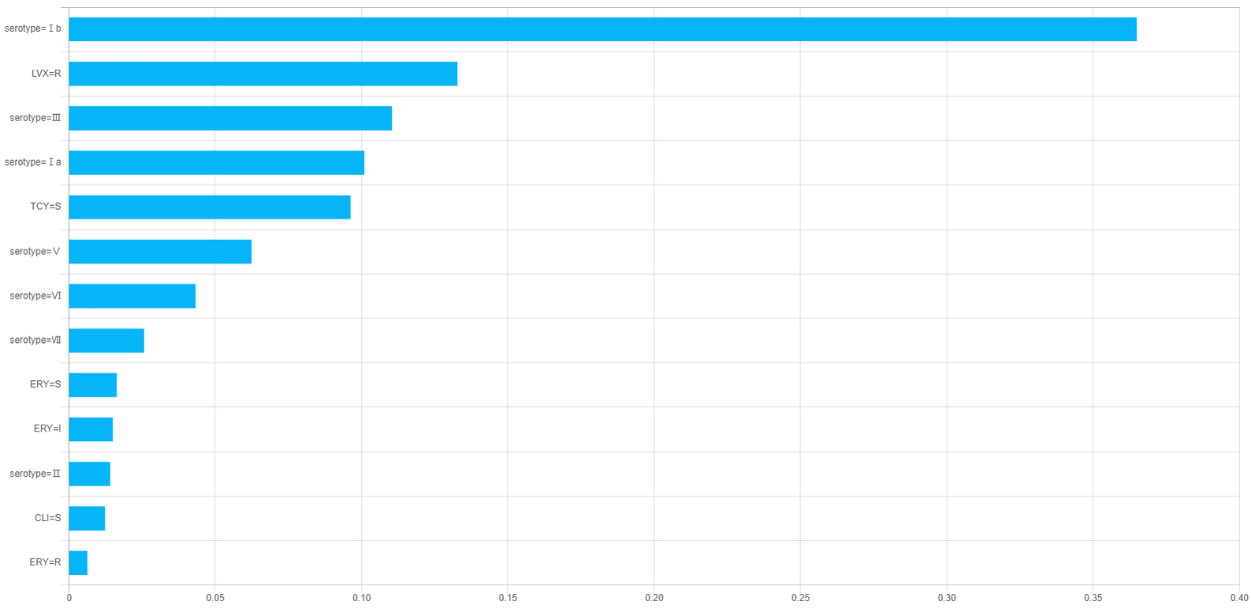


Figure S1 The distribution of feature weights in S&A model. The S&A model was constructed based on antibiotic resistance and serotypes of *S. agalactiae* for clonal complexes prediction*.* Characteristics with higher weight having a greater effect on the model.

Table S1 Comparison of models based on different combination of features in three categories in predicting each CCs of *S. agalactiae*

| CCs | S&A vs V (z, p) | S&A vs S&V (z, p) | S&A vs A&V (z, p) | S&A vs S&A&V (z, p) | V vs S&V (z, p) | V vs A&V (z, p) | V vs S&A&V (z, p) | S&V vs A&V (z, p) | S&V vs S&A&V (z, p) | A&V vs S&A&V (z, p) |
| --- | --- | --- | --- | --- | --- | --- | --- | --- | --- | --- |
| CC1 | -1.784, 0.074 | -2.282, 0.023* | -0.528, 0.597 | -2.338, 0.019* | -0.674, 0.5 | 0.523, 0.601 | -0.867, 0.386 | 0.792, 0.428 | -1.111, 0.266 | -0.876, 0.381 |
| CC10 | -1.049, 0.294 | -1.077, 0.281 | -1.273, 0.203 | -1.299, 0.194 | -0.916, 0.359 | -0.562, 0.574 | -1.304, 0.192 | 0.325, 0.745 | -0.332, 0.74 | -0.855, 0.393 |
| CC12 | 1.386, 0.166 | 0, 1 | 0.995, 0.32 | 0, 1 | -1.386, 0.166 | -0.376, 0.707 | -1.386, 0.166 | 0.995, 0.32 | 0, 1 | -0.995, 0.32 |
| CC17 | 1.665, 0.098 | 1.64, 0.101 | 0.038, 0.969 | 0.038, 0.969 | 0.707, 0.48 | -1.633, 0.102 | -1.633, 0.102 | -1.619, 0.105 | -1.619, 0.105 | 0, 1 |
| CC19 | 0.522, 0.602 | 0.288, 0.773 | -1.199, 0.231 | -1.809, 0.07 | -1.257, 0.209 | -1.076, 0.282 | -1.405, 0.16 | -0.809, 0.418 | -1.169, 0.242 | -1.551, 0.121 |
| CC23 | -1.646, 0.1 | -1.731, 0.083 | -1.653, 0.098 | -1.654, 0.098 | -0.707, 0.48 | -0.707, 0.48 | 0.407, 0.684 | 0.309, 0.757 | 0.813, 0.416 | 0.707, 0.48 |
| CC24 | -1.867, 0.062 | -1.892, 0.059 | -1.959, 0.05 | -1.83, 0.067 | 0.834, 0.404 | 1.013, 0.311 | 0.308, 0.758 | 0.208, 0.835 | -0.803, 0.422 | -0.625, 0.53 |
| Others | -1.891, 0.059 | -1.693, 0.09 | -2.068, 0.039 | -1.851, 0.064 | 0.844, 0.399 | 0.605, 0.545 | 0.72, 0.471 | -0.224, 0.823 | -0.184, 0.854 | 0.126, 0.9 |

- *p<0.05*, the difference is significant. CCs, clonal complexes; A, the parameters of the model include antibiotic resistance only; S&A, serotypes and antibiotic resistance; V, virulence genes detection results only; S&V, serotypes and virulence genes detection results; A&V, antibiotic resistance and virulence genes detection results; S&A&V, serotypes and antibiotic resistance and virulence genes detection results.
